# A Graph Theory Practice on Transformed Image: A Random Image Steganography

**DOI:** 10.1155/2013/464107

**Published:** 2013-12-22

**Authors:** V. Thanikaiselvan, P. Arulmozhivarman, S. Subashanthini, Rengarajan Amirtharajan

**Affiliations:** ^1^School of Electronics Engineering, VIT University, Vellore 632014, India; ^2^School of Information Technology, VIT University, Vellore 632014, India; ^3^School of Electrical & Electronics Engineering, SASTRA University, Thanjavur 613401, India

## Abstract

Modern day information age is enriched with the advanced network communication expertise but unfortunately at the same time encounters infinite security issues when dealing with secret and/or private information. The storage and transmission of the secret information become highly essential and have led to a deluge of research in this field. In this paper, an optimistic effort has been taken to combine graceful graph along with integer wavelet transform (IWT) to implement random image steganography for secure communication. The implementation part begins with the conversion of cover image into wavelet coefficients through IWT and is followed by embedding secret image in the randomly selected coefficients through graph theory. Finally stegoimage is obtained by applying inverse IWT. This method provides a maximum of 44 dB peak signal to noise ratio (PSNR) for 266646 bits. Thus, the proposed method gives high imperceptibility through high PSNR value and high embedding capacity in the cover image due to adaptive embedding scheme and high robustness against blind attack through graph theoretic random selection of coefficients.

## 1. Introduction

Communication has become inevitable in everybody's routine life. Be it mails, texts, photos, or audios or videos, they get communicated in millions among billions. This in turn demands only one thing, that is, security. Information security plays pivotal role to keep the information safe. Among the prominent definitions for information security, the most vital of them is information security which is about veracity, discretion, and data availability. Though several successful methods exist, they are still in research to boost up their performance. Undoubtedly, information security is the soul of exchange of data.

Steganography [1] and cryptography [2] have evolved from times immemorial to provide security for communicating secret details with steganography being the recent one. Image steganography [3–7] is a very interesting field because of the imperceptible way of hiding data due to the resolution of the eye. Image hiding algorithms are used to embed secret data with higher efficiency and less detecting capability [8]. It typically involves encrypting the secret images at first and then embedding the secret information into encrypted images. These in turn have stronger antiattack capability than normal cover image [9].

Image steganography can be done either in spatial domain [10–14] or in frequency domain [15–18]. LSB (least significant bit) embedding [11] is a technique for embedding secret information into a cover image. A mathematical model for LSB technique has been developed for embedding and extracting the secret data [11]. PVD (pixel value differencing) [9, 12] is an efficient technique for spatial domain steganography, which provides high data embedding capacity with reasonable PSNR. PVD is a quite common technique for steganography. Numbers of variations and new methods have been developed for PVD based steganography. The present spatial and transform domain techniques for steganography employ raster scan procedure but are not able to provide high security as data can be extracted easily with blind stegoattack. Solution to this problem is to adopt random steganography [10, 12], which is a new method for escaping from blind stegoattacks because embedding will be done in random manner. This steganography method enhances the security for secret data with the existing steganography algorithms. Pixel indicator method [10] is a random steganography method which is used to select the cover image pixels randomly for embedding and to decide the number of bits to be embedded in the selected pixel. Thus, the robustness of any existing algorithm can be improved by adopting random selection of pixels.

In the transform domain steganography, cover image pixels are converted into coefficients by applying any one of the two-dimensional transforms. Transform coefficients work as a carrier of the secret data in the frequency domain. Three transforms, namely, discrete cosine transform (DCT) [17], discrete wavelet transform (DWT) [16], and IWT [15, 18], are the important transforms for data hiding. The LSB substitution is the broadly used technique in transform based steganography. Studying all the aforementioned available methods, a stable steganography method is preferred to be implemented in IWT domain, which offers better imperceptibility without compromising capacity and security by adapting graceful graph to select the coefficients randomly.

This paper is structured as follows.  Section 2 gives a view about integer wavelet transform and graceful graph is discussed in Section 3.  Section 4 depicts the proposed methodology; Section 5 deals about the results and discussions.  Section 6 demonstrates the steganalysis and finally Section 7 wraps up this work.

## 2. Integer Wavelet Transform

**Figure 1:**
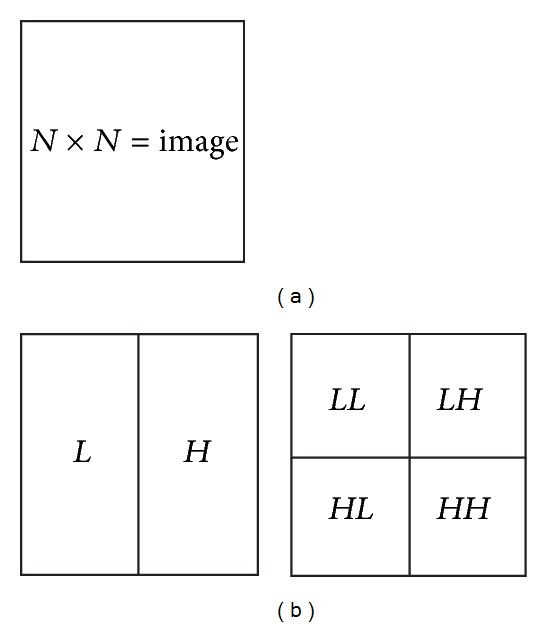
(a) Input image with size *N* × *N*. (b) First level decomposition of the input image.

The following Haar integer wavelet transform (HIWT) can be used to get the coefficients in integer form, and then the LSB substitution for steganography can be achieved by lossless manner. In this paper first level decomposition is adapted which results in approximation (*LL*1), horizontal (*LH*1), vertical (*HL*1), and diagonal (*HH*1) coefficients as shown in Figure 1.

### 2.1. One-Dimensional Decomposition

*Step  1*. Process the image column-wise to get high pass filtered output (*H*1) and low pass filtered output (*L*1). The size of *H*1 and *L*1 is *N* × *N*/2

(1) $$\begin{array}{c} H1=\left(C_{o}-C_{e}\right),\\ L1=\left(C_{e}+\text{Floor}\left(\frac{H}{2}\right)\right), \end{array}$$

where *C*_*o*_ and *C*_*e*_ are the odd column and even column-wise pixel values.

### 2.2. Two-Dimensional Decomposition

*Step  2*. Take *H*1 and *L*1 for getting the approximation (*LL*1), horizontal (*LH*1), vertical (*HL*1), and diagonal (*HH*1) coefficients. Size of all four sets of coefficient is *N*/2 × *N*/2. Row-wise processing will be adapted here on *H*1 and *L*1. Separate the odd and even rows of *H*1 and *L*1 as follows:

 
*H*_odd_—odd row of *H*1,
 
*L*_odd_—odd row of *L*1,
 
*H*_even_—even row of *H*1,
 
*L*_even_—even row of *L*1.

The following equations are used to get the 2D integer wavelet transform:

(2) $$\begin{array}{c} LH1=L_{\text{odd}}-L_{\text{even}},\\ LL1=L_{\text{even}}+\text{Floor}\left(\frac{LH}{2}\right),\\ HH1=H_{\text{odd}}-H_{\text{even}},\\ HL1=H_{\text{even}}+\text{Floor}\left(\frac{HH}{2}\right). \end{array}$$

## 3. Graceful Graph for Random Path

Graph theory is the study of points and lines. In particular, it involves the ways in which the sets of points called vertices (*V*) can be connected by lines or arcs called edges (*E*). Any graph can be represented as *G*(*V*, *E*). In the steganographic point of view, pixels or coefficients are considered as nodes and connection between two nodes is called edge. Graceful graphs play an important role for random traversing in steganography algorithms.

### 3.1. Procedure for Graph Generation

Since in the steganographic point of view the coefficients are considered as nodes and given the number of nodes (*N*), following four sequences *S*1, *S*2, *S*3, and *S*4 can be formed as follows:

(3) $$\begin{array}{c} S1=\left[2q,2q-1,2q-2,\ldots ,q+1\right], \end{array}$$

where “*q*” is an integer and calculated as *q* = *N*/4. *N* represents total number of nodes. *N* should be multiple of 4:

(4) $$\begin{array}{c} S2=\left[q_{s}\left(1\right)+\sigma \left(1\right),q_{s}\left(2\right)+\sigma \left(2\right),\ldots ,q_{s}\left(q\right)+\sigma \left(q\right)\right], \end{array}$$

where *q*_*s*_(*i*) is *i*th element of the sequence {*q*, *q* − 1, *q* − 2, *q* − 3,…, (*q* − *N*/4) + 1}, *σ*(*i*) is *i*th element of the sequence (*σ*) obtained by random arrangement of the elements of “*q*_*s*_.”

Sequence *S*3 is formed with the elements of “*σ*” as

(5) $$\begin{array}{c} S3=\left[\sigma \left(q\right),\sigma \left(q-1\right),\ldots ,\sigma \left(1\right)\right]. \end{array}$$

*S*4 is a sequence containing “*q*” zeros

(6) $$\begin{array}{c} S4=\left[0,0,0,\ldots \right]. \end{array}$$

The previous sequences are concatenated to form a new sequence “*S*”:

(7) $$\begin{array}{c} S=\left[\begin{array}{cccc} S1 & S2 & S3 & S4 \end{array}\right]. \end{array}$$

A graceful graph table is generated by using node sequence (NS), edge numbers (*E*), sequence “*S*”, and a sequence “*A*” (containing sum of elements of *E* and *S*). Graph will be generated using this table. The entire graph generation is illustrated in the example given below.

### 3.2. Example for a Graph Generation

A simple example is taken for explanation; this graph is generated by considering a 4 × 4 matrix containing 16 elements. Therefore, *N* = 16.

*Step 1*. Generation of sequence *S*1: consider an integer “*q*”, is calculated by *q* = *N*/4. Since *N* is 16, *q* = 4. *S*1 is generated using (3):

(8) $$\begin{array}{c} S1=\left[\begin{array}{cccc} 8 & 7 & 6 & 5 \end{array}\right]. \end{array}$$

*Step  2*. Generation of sequence *S*2: using (4), *q*_*s*_ = [4  3  2  1], *σ* = [1  4  3  2] is a permuted sequence of *q*_*s*_:

(9) $$\begin{array}{c} S2=\left[\begin{array}{cccc} 5 & 7 & 5 & 3 \end{array}\right]. \end{array}$$

*Step  3*. Generation of sequence *S*3: substituting *σ* = [1  4  3  2] in (5),

(10) $$\begin{array}{c} S3=\left[\begin{array}{cccc} 2 & 3 & 4 & 1 \end{array}\right]. \end{array}$$

*Step  4*. Generation of the sequence *S*4: since *q* = 4,

(11) $$\begin{array}{c} S4=\left[\begin{array}{cccc} 0 & 0 & 0 & 0 \end{array}\right]. \end{array}$$

*Step  5*. Generation of sequence *S*:

(12) $$\begin{array}{c} S=\left[\begin{array}{cccccccccccccccc} 8 & 7 & 6 & 5 & 5 & 7 & 5 & 3 & 2 & 3 & 4 & 1 & 0 & 0 & 0 & 0 \end{array}\right]. \end{array}$$

*Step  6*. Generation of a graceful graph table: Table 1 shows graceful graph table for random co-efficient selection.

Steps to generate graceful graph table are as follows.

*Step  6.1*. Generate the sequence NS based on the number of nodes (*N*): NS = {1,2,…, *N*}.

*Step  6.2*. Generate the edge sequence *E* = {1,2,…, *N* − 1} where the first element of sequence *E* will fall below second element of the sequence NS and so on.

*Step  6.3*. Arrange the *S* sequence in Table 1 and remove the first element of *S*.

*Step  6.4*. Compute the final sequence *A* by adding the elements of *E* and (*A* = *E* + *S*). 

*Step  7*. Draw a graceful graph by considering the sequences *S* and *A*. 

**Figure 2:**
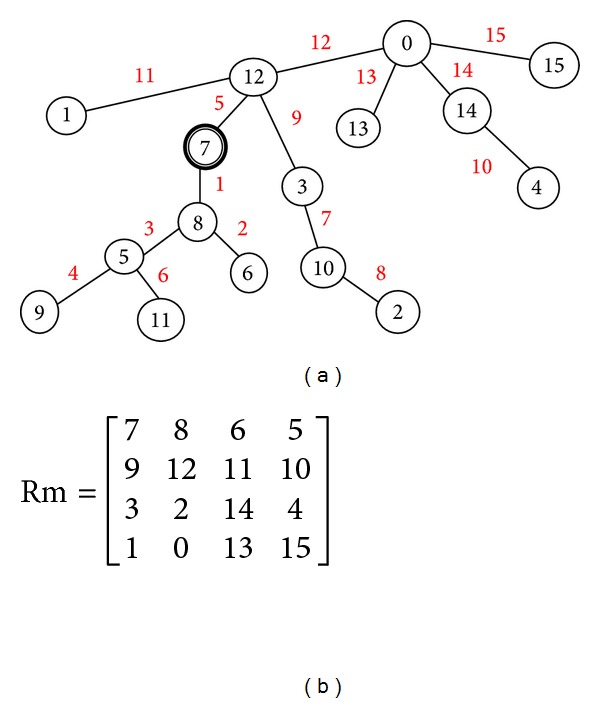
(a) Graceful graph. (b) Rm matrix.

The elements of *S* and *A* are the nodes for the graph and are scanned in a zigzag manner as shown in Table 1 with another colour. Connection between the nodes is the edge with difference of the node numbers being the label for the edge. The graph generated is shown in Figure 2(a). A 4 × 4 matrix is generated by arranging the nodes in the increasing order of the labels. Repeating nodes will be discarded while generating the matrix; that is, the matrix should contain one node only once. The generated matrix is named as “Rm” (random matrix) and shown in Figure 2(b). This Rm is considered as key 2 in this methodology. In the matrix “Rm” element 0 represents the position of the co-efficient to be embedded first and 15 represents the position of the co-efficient to be embedded at last.

## 4. Proposed Methodology

**Figure 3:**
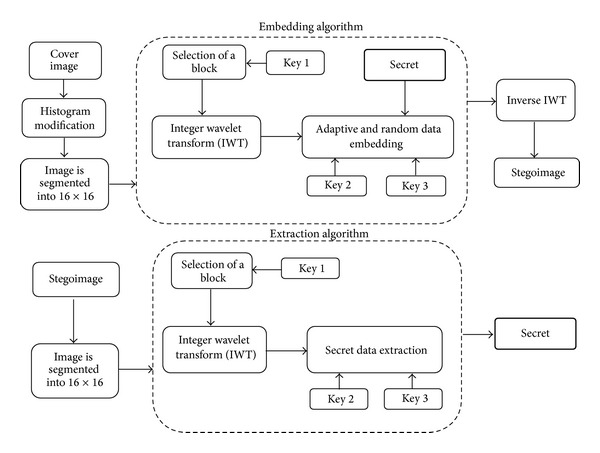
Block diagram of proposed methodology.

Figure 3 shows the proposed methodology for highly random and robust steganography. First the cover image is given to histogram modification section to change the pixel values between 15 and 240. Then we segment the cover image into 16 × 16 nonoverlapping blocks and apply the integer wavelet transform. Then key 1 is used for selecting a particular 16 × 16 block, key 2 generated using graceful graphs is used to select the coefficients, and key 3 is the number of bits to be embedded in the selected coefficients. Finally, inverse IWT is applied to construct the stegoimage.

### 4.1. Embedding Algorithm

*Step  1*. Consider a 512 × 512 grayscale image as the cover image (CI); then

(13) $$\begin{array}{c} \text{C}{\text{I}}_{ij}\in \left\{0,1,2,3,\ldots ,255\right\}, \end{array}$$

where *i* and *j* are varying from 1 to 512.

*Step  2*. Generate a secret data (SD). Here a stream of binary data is considered as secret data:

(14) $$\begin{array}{c} \text{SD}\in \left\{0,1\right\}. \end{array}$$

*Step  3*. Apply histogram modification on the cover image to restrict the pixel values between 15 and 240. Now cover image is denoted as CI′:

(15) $$\begin{array}{c} {\text{CI}}_{i,j}^{\prime }\in \left\{15,16,17,\ldots ,240\right\}. \end{array}$$

*Step  4*. Segment the image into 16 × 16 sized blocks.

*Step  5*. Read all the 16 × 16 blocks one by one from top to bottom and assign numbers to all the blocks as per raster scan procedure.

*Step  6*. Generate a random number sequence (RS) that contains unique positive integers between 1 and 1024 with the length 1024 (because the cover image is segmented into 1024 blocks of 16 × 16 size). This sequence is used to select the 16 × 16 blocks randomly. This is considered as Key1. If the first element of RS is 3 then the 3rd block will be selected first for embedding

(16) $$\begin{array}{c} \text{RS}\in \left\{1,2,3,\ldots ,255,\ldots ,1024\right\}. \end{array}$$

*Step  7*. Apply Haar integer wavelet transform to the selected 16 × 16 block. It will result in four subbands as approximation (*LL*1), horizontal (*LH*1), vertical (*HL*1), and diagonal (*HH*1) coefficients (*C*) with the size of 8 × 8. In this methodology, embedding will be done in all subbands except *LL*1 subband to maintain good imperceptibility.

*Step  8*. Three different Rm matrices (Key-2) will be generated with *N* = 64 using graceful graph corresponding to *LH*1, *HL*1, and *HH*1, subbands. Those matrices will be used to select the coefficients randomly in each subband.

*Step  9*. Adaptive bit embedding procedure is adopted and the number of bits to be embedded in the selected co-efficient is given by “*L*” (bit length) in (17). This is considered as key 3. Modulus of *C* is considered in case it holds negative value. Consider

(17) $$\begin{array}{c} L=\{\begin{array}{cc} 4, & C\geq 16,\\ 3, & \text{if}\;8\leq C<16,\\ 2, & \text{if}\;4\leq C<8,\\ 1, & \text{if}\;C<4. \end{array} \end{array}$$

*Step  10*. *L* numbers of bits are taken from SD and embedded by using LSB [11] embedding procedure on the selected co-efficient (*C*). Repeat this procedure for all the *C* values and the remaining subbands. 

*Step  11*. Repeat Steps 7 to 10 for all the 16 × 16 blocks. 

*Step  12*. Apply inverse integer wavelet transform to each 16 × 16 block and produce the stegoimage (SI), SI_*ij*_ ∈ {0,1, 2,3,…, 255}, where *i* and *j* are varying from 1 to 512.

### 4.2. Extraction Algorithm

Exact reverse procedure should be used to extract the secret data. 

*Step  1*. Get 512 × 512 stegoimage SI_*ij*_ ∈ {0,1, 2,3,…, 255}.

*Step  2*. Segment the image into 16 × 16 sized blocks.

*Step  3*. Use key 1 to select the 16 × 16 block.

*Step  5*. Apply integer wavelet transform to the selected 16 × 16 block. This will result in approximation (*LL*1), horizontal (*LH*1), vertical (*HL*1), and diagonal (*HH*1) coefficients. 

*Step  6*. Use key 2 for random selection of coefficients in each subband. 

*Step  7*. Use key 3 for calculating the number of bits to be extracted [11] from the selected co-efficient. 

*Step  8*. Repeat the above procedure for all the *C* values and all 16 × 16 sized blocks.

## 5. Results and Discussion

Proposed method has been evaluated with seven images with the size of 512 × 512. The performance characteristics are evaluated through MSE (mean squared error) and peak signal to noise ratio (PSNR). PSNR is calculated by using the following equation (18), where *M* and *N* are the sizes of the given image:

(18) $$\begin{array}{c} \text{MSE}=\frac{1}{MN}\overset{M}{\underset{i=1}{\sum }}\overset{N}{\underset{j=1}{\sum }}{\left(C_{i,j}-S_{i,j}\right)}^{2},\\ \text{PSNR}=10\;\log_{10}\frac{255*255}{\text{MSE}}\text{dB}. \end{array}$$

**Figure 4:**
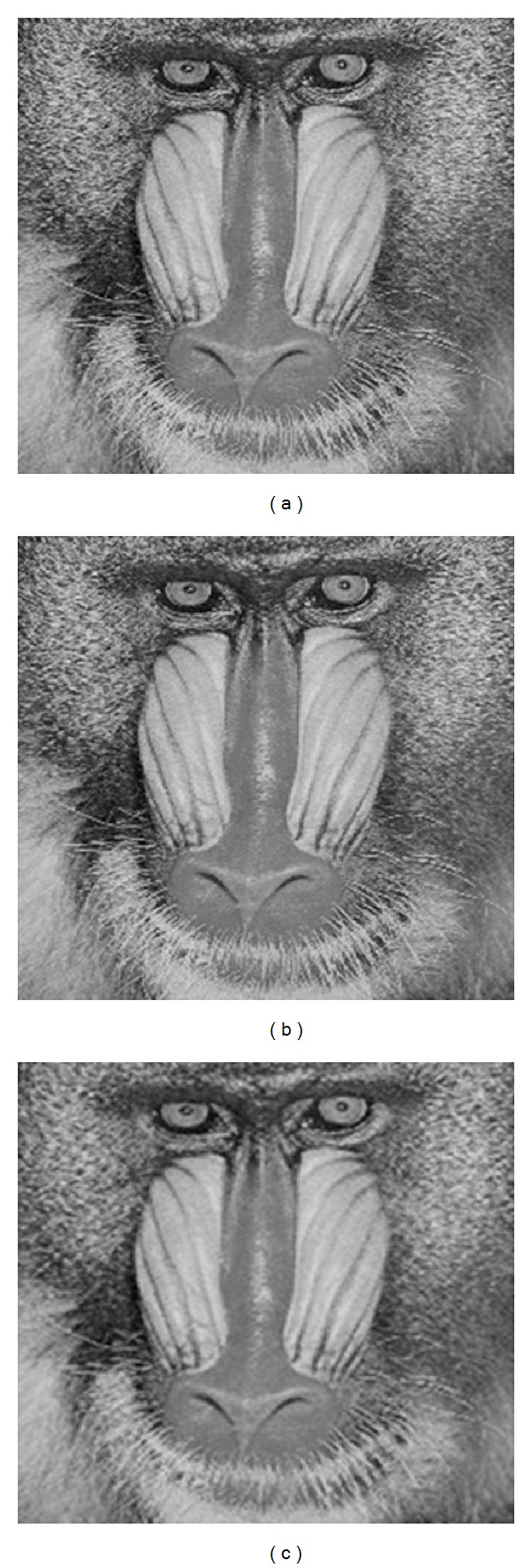
(a) cover image, baboon. (b) Stegoimage with *HH*1 embedding. (c) Stegoimage with *LH*1, *HL*1, and *HH*1 subband.

Cover image is divided into 16 × 16 nonoverlapping blocks. Therefore, wavelet subband size is 8 × 8. Here, embedding is done with two different ways involving the embedding in one subband and embedding in all the subbands except *LL*1. Key 1, key 2, and key 3 provide high security against blind steganography attacks or human visual attacks. All the results are tabulated in Table 2. For Lena image PSNR value is around 49 dB, 47 dB, and 56 dB when the data is embedded only in *LH*1, *HL*1, and *HH*1, respectively. Embedding only in *HH*1 subband resulted in PSNR above 50 dB. Maximum of 144540 bits are embedded in Barbara image with embedding only in *HL*1. Technique of embedding data in only one subband shows high imperceptibility but less embedding capacity. However, the technique of embedding data in all the subbands except *LL*1 results in increased number of bits with reasonable PSNR value around 44 dB, thus reflecting the advantage of IWT domain steganography. Figures 4(a), 4(b), and 4(c) show cover image, baboon, stegoimage with HH1 embedding, and stegoimage with 3-subband embedding. All the images are looking the same; therefore, this technique can escape from visual attack.

## 6. Steganalysis

Steganalysis is the blind inspection of embedded data in stegoimage. This proposed method is highly robust against the blind attacks. This method is a transform domain method, so that secret data cannot be extracted from the spatial domain. Key 1, key 2, and key 3 impart high randomness for embedding. The following numbers of iterations are required to extract the hidden information from the stegoimage generated by the proposed method:

(19) Total  number  of  iterations  (TI)  =(1024!∗3!∗64!∗4∗64∗3)∗1024,

where 1024! is the possible order of traversals among 1024 numbers of 16 × 16 sized blocks, 3! gives the possible order of the subbands in which the data is embedded, 64! represents different Rm matrices, 4 represents the maximum bit length. 64 represents the total number of coefficients in each subband, 3 represents the total number of subbands, and 1024 represents the total number of 16 × 16 blocks.

The proposed methodology is compared with the existing techniques and the TI values are tabulated in Table 3. Thus, the large difference observed in the total number of iterations required for the proposed technique and the existing technique clearly elucidates the enhanced data security against blind attacks.

## 7. Conclusion

In the proposed work, IWT along with graceful graph offers a secure and random image steganography with high imperceptibility. Moreover, this adaptive random embedding process results in high capacity and robustness against blind attacks. Higher imperceptibility is attained when the data is embedded only in one subband, whereas higher capacity with reasonable PSNR is observed when the data is embedded in all the subbands except *LL*1 subband. This proposed method can be enhanced by using different integer transforms to attain higher PSNR and robustness can be improved further by adding multiple security methods.

## Figures and Tables

**Table 1:** Graceful graph table.

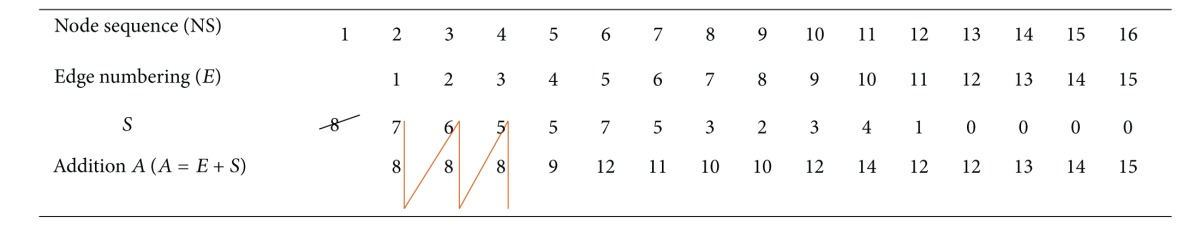

**Table 2:** Results for various images.

Image	Embedding only in LH1		Embedding only in HL1		Embedding only in HH1		Embedding in LH1, HL1, and HH1 subbands	
	PSNR (dB)	Total bits	PSNR (dB)	Total bits	PSNR (dB)	Total bits	PSNR (dB)	Total bits
Lena	49.3802	85421	47.4721	98978	56.3353	71795	44.9682	256174
Baboon	44.4302	135275	44.5608	135123	54.0215	93587	41.315	363965
Cameraman	47.857	94815	47.3303	95353	55.4215	76498	44.2326	266646
Taj Mahal	46.8793	101949	46.8283	101515	55.9065	74440	43.6245	277884
Peppers	48.4761	90894	47.7119	95704	56.4168	71014	44.8319	257592
Barbara	45.3901	117307	42.8743	144540	49.1536	137097	40.3423	398924
Airplane	47.0608	99695	47.321	96611	55.7802	75529	43.9919	271815

**Table 3:** Comparative analysis.

Method	Total number of iterations to extract data (TI)
Proposed method	(1024! ∗ 3! ∗ 64! ∗ 4 ∗ 64 ∗ 3) ∗ 1024
Fazli et al. [16]	TI = 256 ∗ 256 ∗ 3
Song et al. [17]	TI = 64! ∗ 64
Sakkara et al. [18]	TI = 16! ∗ 64 ∗ 64 ∗ 3
